# Supplementary material for: TP53 mutation screening for patients at risk of myeloid malignancy
Source: medRxiv. 2024 Feb 8:2024.02.06.24302401. Preprint. [Version 1] doi: 10.1101/2024.02.06.24302401 (PMC10896414; doi:10.1101/2024.02.06.24302401)
Supplement: Supplement 1 [file media-1.pdf]

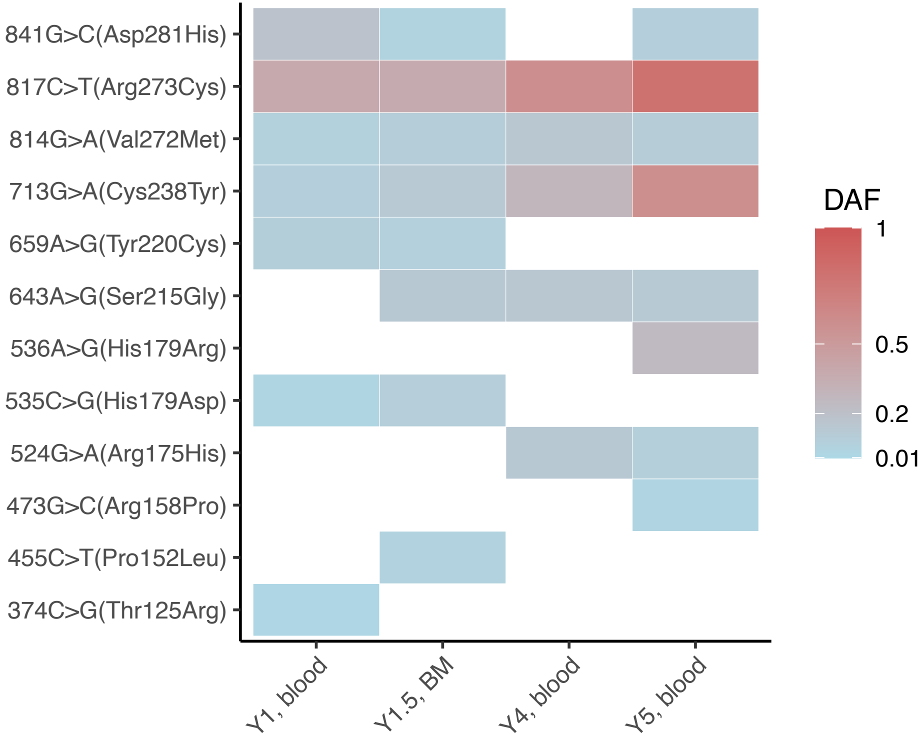

Supplementary Figure 1. Variant allele frequency (VAF) kinetics for the detected variants in patient 2.
